# Eye and hair color prediction of an early medieval adult and subadult skeleton using massive parallel sequencing technology

**DOI:** 10.1007/s00414-023-03032-y

**Published:** 2023-06-07

**Authors:** Irena Zupanič Pajnič, Tamara Leskovar, Matija Črešnar

**Affiliations:** 1 Institute of Forensic Medicine, Faculty of Medicine, University of Ljubljana, Korytkova 2, 1000 Ljubljana, Slovenia; 2 Centre for Interdisciplinary Research in Archaeology, Department of Archaeology, Faculty of Arts, University of Ljubljana, Ljubljana, Slovenia

**Keywords:** Skeletal remains, Ancient DNA, Eye color, Hair color, HIrisPlex, Petrous bones

## Abstract

Phenotypic trait prediction in ancient DNA analysis can provide information about the external appearance of individuals from past human populations. Some studies predicting eye and hair color in ancient adult skeletons have been published, but not for ancient subadult skeletons, which are more prone to decay. In this study, eye and hair color were predicted for an early medieval adult skeleton and a subadult skeleton that was anthropologically characterized as a middle-aged man and a subadult of unknown sex about 6 years old. When processing the petrous bones, precautions were taken to prevent contamination with modern DNA. The MillMix tissue homogenizer was used for grinding, 0.5 g of bone powder was decalcified, and DNA was purified in Biorobot EZ1. The PowerQuant System was used for quantification and a customized version of the HIrisPlex panel for massive parallel sequencing (MPS) analysis. Library preparation and templating were performed on the HID Ion Chef Instrument and sequencing on the Ion GeneStudio S5 System. Up to 21 ng DNA/g of powder was obtained from ancient petrous bones. Clean negative controls and no matches with elimination database profiles confirmed no contamination issue. Brown eyes and dark brown or black hair were predicted for the adult skeleton and blue eyes and brown or dark brown hair for the subadult skeleton. The MPS analysis results obtained proved that it is possible to predict hair and eye color not only for an adult from the Early Middle Ages, but also for a subadult skeleton dating to this period.

**Supplementary information:**

The online version contains supplementary material available at 10.1007/s00414-023-03032-y.

## Introduction

Improvements in forensic genetic analysis using massive parallel sequencing (MPS) technology [4, 8, 25] have introduced a powerful tool for phenotypic trait prediction of skeletal remains. This can be used not only in a forensic context [6, 16, 40, 44, 48, 74, 78] but also in ancient DNA (aDNA) analysis to obtain information about externally visible characteristics of individuals from past human populations. In forensics, phenotypic traits associated with pigmentation, such as eye, hair, and skin color, were introduced in the past [38, 39, 77], and the HIrisPlex and HIrisPlex-S systems were developed and are used together with genotype and phenotype databases established for pigmentation prediction [79]. Even if aDNA is poorly preserved, and if it shows low DNA quantity and quality, high susceptibility for contamination, and the presence of inhibitors [41], several studies have successfully predicted pigmentation of ancient skeletons. With comparison of predicted colors and historical documentation, a Polish general was positively verified [16], and some other historical questions have been solved [29, 58]. Pigmentation traits were predicted for the remains of King Richard III of England [42], and even for skeletons several hundred years old, it has been possible to obtain information about what people’s ancestors looked like [83]. However, in the literature reviewed, eye and hair color prediction was established on ancient adult skeletons, but it was not possible to find data on eye and hair color prediction for ancient subadults. It is known that extrinsic factors affect subadult skeletons more than adult ones [3, 31, 52, 66], and, in comparison to adult skeletons, subadult skeletons are more susceptible to loss and decay [14, 27, 50, 75, 76, 80], resulting in low DNA preservation. Some studies have shown that DNA is better preserved in ancient adult skeletons than in subadult skeletons, and differences have been identified in the bone characteristics of adults and subadults [73]. We therefore wondered whether it is possible to determine hair and eye color not only for ancient adult skeletons, but also for subadult skeletons using MPS technology. To verify this, an adult skeleton and a subadult skeleton excavated from the same grave from the Early Middle Ages were selected for analysis, ensuring that DNA preservation was influenced by similar environmental factors for the same amount of time.

For successful aDNA analysis, the most suitable type of bone and its intra-bone part has to be selected because of high differences in preservation of DNA in different skeletal element types [1, 17, 18, 23, 32, 34, 49, 51, 53, 82]. Various studies have shown that the petrous bone performs best in aDNA analyses because of its hard and dense tissue (the petrous bone is considered the hardest and densest of all the bones in the body), resulting in better protection and higher resistance of DNA to difficult environmental conditions [11, 21, 22, 28, 30, 56, 59, 60, 68]. Accordingly, petrous bones were used for eye and hair color prediction of the adult and subadult early medieval skeletons in this study.

Approval from the Slovenian Medical Ethics Committee was obtained for this study (145/06/13, 0120–412/2021/3), and the persons included in the elimination database signed an informed consent statement.

## Material and methods

### Selection of bone samples

In 1974, excavation of skeletal remains from the Bled–Pristava archaeological site was performed under the leadership of Timotej Knific from the Ljubljana National Museum, and the remains were stored in the museum warehouse. The Bled–Pristava archaeological site is located in northern Slovenia, near Bled. It is a multiperiod necropolis that was active from the third to ninth centuries AD. The individuals buried there are partially indigenous people (third to sixth century) and partially Slavs that migrated to the area. A unique feature of this necropolis is a multiple burial grave, which was separate from the other graves, located on the edge of the necropolis. Four individuals were excavated from this multiple grave and, based on stratigraphy, they were all placed in the grave at the same time, indicating their simultaneous deaths. Because of the uniqueness of the finding, kinship analysis of the four skeletons has been performed, and a father–children kinship relationship has been proven for the excavated skeletons [88]. Because genetic studies of younger subadults (especially below age 3) are the most challenging [27] due to higher susceptibility to decay, as a result of low tensile and compressive strength of immature bones [14], the youngest subadult skeleton from the multiple burial grave was selected for a phenotyping study. In addition to the youngest subadult skeleton, the oldest adult skeleton was selected for eye and hair color prediction. Both skeletons (the father and the youngest child) were dated to the fifth to sixth century AD. Based on anthropological analysis, the adult skeleton was characterized as a middle-aged man (between 35 and 45 years old). For the subadult skeleton, it was not possible to determine sex anthropologically, and the age was estimated to be around 6 years. The right petrous bone was sampled from each skeleton. To consider intra-bone variability in DNA content, the densest portion—the cochlea, in which DNA is preserved the best—was used for genetic analysis. The cochlea was detached from the rest of the petrous bone following a detailed protocol constructed by Pinhasi et al. [61]. To build an elimination database, we collected buccal swabs from every individual that had had recent contact with the skeletons studied and from every individual that took part in the anthropological and genetic investigations.

### Preventing contamination

The very high risk of contamination with modern DNA limits the success of aDNA analysis [65], and so a number of precautions were followed to prevent contamination and to verify ancient DNA authenticity [12, 20, 55, 64]. Extraction of DNA from ancient petrous bones was performed in a special room designed for processing ancient skeletons only, and all the work was done in a closed MC 3 microbiological safety cabinet (Iskra Pio, Šentjernej, Slovenia) outfitted with UV light and a HEPA filter [84]. All the tools used for drilling, cutting, and grinding were cleaned with bleach, water, and ethanol, sterilized in a Europa B xp sterilizer (Tecno-Gaz, Parma, Italy), and UV irradiated for 30 min in a BLX-Multichannel BioLink DNA Crosslinker (Vilber, Collégien, France). The laboratory plastics and reagents (except those labeled DNase-free or DNA-free) were also sterilized and UV irradiated before use. For contamination monitoring, extraction-negative controls (ENCs) were processed together with the bone samples [57], and all individuals involved in the analyses were typed, and their genotypes were entered into the elimination database. Various purification machines were used for purifying low-DNA-template bone samples (the EZ1 Advanced XL biorobot,Qiagen, Hilden, Germany) and high-DNA-template elimination database samples (the BioRobot EZ1 machine; Qiagen).

### DNA extraction

All samples were mechanically and chemically cleaned in order to eliminate surface contamination [84]. Chemical cleaning consisted of 5% Alconox (Sigma-Aldrich, St. Louis, MO, USA), 80% ethanol (Merck, Kenilworth, NJ, USA), and sterile bi-distilled water (Sartorius-Stedim Biotech, Göttingen, Germany). After processing, the bones were dried and then cooled with liquid nitrogen. Cooled bones were cut with a sterilized diamond saw (Schick, Schemmerhofen, Germany). We exposed the bones to UV light for 30 min with a BLX-Multichannel BioLink DNA Crosslinker (Vilber) before grinding them. A Bead Beater MillMix 20 homogenizer (Tehtnica, Domel, Železniki, Slovenia) was used for grinding, which produced a fine homogenous powder. Petrous bones were used to extract DNA from 500 mg of powder following the extraction protocol reported by Zupanič Pajnič [84], with decalcification with 0.5 M of ethylenediaminetetraacetic acid, or EDTA (Sigma-Aldrich), and purification was carried out with the EZ1 DNA Investigator Kit (Qiagen) and EZ1 Advanced XL machine (Qiagen), selecting trace protocol, TE buffer elution, and 50 µl of final volume option as reported by Zupanič Pajnič [84]. We performed duplicated DNA extractions from petrous bones, obtaining extract 1 and extract 2 from each petrous bone, and ENC samples were added to each extraction batch to monitor the purity of the reagents and plastics used for extraction of DNA.

### DNA quantification

Determination of the concentration of human nuclear DNA (short 85 bp autosomal fragment, Auto target), male DNA (Y target), and long 294 bp autosomal fragment (Deg target) was achieved using the PowerQuant System (Promega), according to the technical manual [62]. The results calculated for the Auto and Deg targets were used to calculate the degradation index (the Auto/Deg ratio), and we detected the PCR inhibitors using an internal PCR control (IPC shift). Quantification was performed using the QuantStudio 5 Real-Time PCR system, Quant-Studio Design and Analysis Software 1. 5. 1 (Applied Biosystems, AB, Foster City, CA, USA), and the PowerQuant Analysis Tool software (https://worldwide.promega.com/resources/tools/powerquant-analysis-tool/).

To lower the variability of results, real-time quantification was carried out in duplicate [62]. The efficiency of extraction was displayed in DNA quantity obtained from 1 g of bone and expressed in ng DNA per gram of bone powder; calculation was performed from the PowerQuant Auto target results.

### STR typing

Short tandem repeat (STR) typing was performed for authentication of ancient samples and comparison with the elimination database. Together with the positive control (9948-Qiagen), the petrous bone and elimination database samples, PCR negative control, and ENCs were typed to exclude any possibility of contamination with modern DNA during DNA isolation. Two extracts were obtained from each petrous bone, and both of them were typed for autosomal STR markers. The Investigator EssplexPlus SE QS kit was used for typing, using 30 amplification cycles and a 25-μl reaction volume following the manufacturer’s protocols [63]. One nanogram of bone DNA (calculated from the PowerQuant Auto target) and positive control DNA were used as a template, and the maximum volume of DNA extract was used for amplification of ENCs. PCR amplification was run in the Nexus Master Cycler (Eppendorf, Hamburg, Germany, EU). In addition, positive and negative PCR controls were amplified. The SeqStudio Genetic Analyzer for HID (Thermo Fisher Scientific, TFS, Carlsbad, CA, USA) [71], SeqStudio Data Collection Software v 1.2.1 (TFS), and GeneMapper ID-X Software v 1.6 (TFS) were used for generation of genetic profiles. Together with monitoring the cleanliness of ENC samples, differentiation of the skeletons’ profiles and the profiles obtained from the elimination database makes it possible to authenticate aDNA.

For calculating the frequency of the adult’s and subadult’s STR genotype, composite profiles were constructed from the duplicated analysis [7], and DNA VIEW software version 37.37 [5] was used, employing allele frequencies of the modern Slovenian population [85–87].

### SNP typing with the HIrisPlex panel

A customized HIrisPlex panel for amplification of 24 SNPs using 17 pairs of PCR primers [6, 44] was used for pigmentation prediction with the MPS technology for the extracts obtained in the second round of extraction (extract 2) from the adult and subadult petrous bone. Analysis was performed on the Ion S5 System (TFS). One nanogram of DNA (calculated from the PowerQuant Auto target) was used for library preparation for the adult and subadult skeleton extracts, following the manufacturer’s recommendations [71]. For target amplification, 25 cycles were used. Library preparation and templating were fully automated using the Ion Chef System (TFS). The Precision ID DL8 Kit for Ion Chef was used for library preparation following the manufacturer’s instructions [71]. The concentration of the combined library pools was determined in duplicate along with standards and negative controls with the Ion Library TaqMan Quantification Kit (TFS) according to the manufacturer’s recommendations [72]. The library pool, which included amplicons from the adult and subadult skeleton from the fifth to sixth century, was diluted to 30 pM. The template was loaded onto an Ion 530 sequencing chip (TFS) in a single chip loading workflow. For sequencing, Ion S5 Precision ID sequencing reagents (TFS) and Ion S5 Precision ID sequencing solutions (TFS) were used. Ion Torrent™ Suit Software 5.6 (TFS) was used to align reads against the *Homo sapiens* reference genome (hg19). The Coverage Analysis v 5.6.0.1. plugin was used to obtain data on the sequence coverage level in targeted regions. The default settings for genotyping were applied according to the manufacturer’s recommendations [72] using Converge™ software version 2.0 (TFS).

For the HIrisPlex eye and hair color prediction of the adult and subadult skeletons from the fifth to sixth century, the R-script program (https://walshlab.sitehost.iu.edu) was used, and the skeletons’ genotypes were converted into the corresponding input codes, and these were uploaded into the HIrisPlex web tool (accessible at https://hirisplex.erasmusmc.nl). The most likely eye and hair color prediction based on the *p*-value and AUC (area under the receiver operating curve) loss was used to predict eye and hair color.

## Results

### DNA quantification

Real-time PowerQuant results (Auto, Deg, and Y targets (calculated as ng DNA/µl of extract), IPC shift, and Auto/Deg ratio) for all of the extracts acquired from petrous bones and ENCs processed in combination with the samples analyzed are presented in Supplementary material, SM 1. Because amplifications were performed in duplicate, average measurements are reported here. For each petrous bone, we present the quantity of DNA per 1 g of powder and express it in ng DNA/g of powder. The petrous bone extracts yielded a high amount of DNA (on average, 17.4 ng DNA/g of the adult petrous bone and 16.6 ng DNA/g of the subadult petrous bone). Amplifying the Y PowerQuant targets confirmed the presence of male DNA in both petrous bones, and male sex was additionally confirmed with amplification of the Y chromosome amelogenin locus included in the STR typing amplification kit (see SM 2).

We observed a high degradation index in both skeletons; the average value was 276 for the adult petrous bone and 53 for the subadult petrous bone (see SM 1). The values of the IPC shift were less than 0.30 (threshold recommended by the manufacturer; [62]) in all extracts obtained from the adult and subadult petrous bone, indicating efficient DNA purification (see SM 1). Considering the ENC employed, no PowerQuant targets were detected, except for ENC 2, where Auto target amplification resulted in a measurement of 0.0002 ng DNA/µl, as can be seen in SM 1. When the PowerQuant System validation study was performed, it was shown that a concentration of DNA as small as 0.0005 ng DNA/µl can be detected with certainty [19], and the Auto target measurement of ENC 2 was lower. However, as expected, no STR genetic profiles were obtained from any ENCs, indicating no presence of contamination originating from the extraction process.

### STR typing

For authentication of ancient samples, autosomal STRs were typed and compared with elimination database profiles. Out of 16 STR loci and the amelogenin gene, 13 STR loci and the amelogenin locus were successfully amplified in the adult skeleton, 15 STR loci and the amelogenin gene were successfully typed in the subadult skeleton, and no match was found with elimination database profiles. No profiles were obtained from the ENC samples and the PCR negative control sample. STR profile of PCR-positive control matched the profile of 9948 DNA [63]. The autosomal STR profiles of the adult and subadult skeleton from the fifth to sixth century acquired using the Investigator Essplex SE QS kit (Qiagen) are presented in SM 2. Although an optimal quantity of DNA (1 ng) was amplified in the petrous bones, there were allelic drop-out events. The explanation for this lies in the severe degradation of DNA that is isolated from fifth- to sixth-century skeletons. Specifically, allele drop-outs were identified at high-molecular-weight alleles at the D8S1179, D21S11, and D2S1338 loci in the adult skeleton and at D2S1338 in the subadult skeleton. All dropped-out loci were the longest STR loci in the Investigator EssplexPlus SE QS kit (Qiagen). Allelic drop-out events frequently occur with ancient samples, and high degradation has an effect on the allelic drop-out phenomenon, in particular at loci with a high molecular weight, which are more susceptible to drop-outs due to less efficient PCR, more frequent stuttering, and more evident degradation effects [7]. The genotype frequencies of the adult and subadult skeleton calculated for composite profiles employing allele frequencies of the modern Slovenian population [85–87] were 2.2 × 10^−17^ for the adult skeleton and 1.04 × 10^–20^ for the subadult skeleton. Because allele frequencies of ancient populations that inhabited Slovenia in the past are not available, comparison of genotype frequencies with the modern Slovenian population was not possible.

### HIrisPlex SNP typing and prediction of eye and hair color

Quantification results obtained using the Ion Library TaqMan Quantification Kit (TFS) indicated that the library pool including extracts obtained from the adult and subadult ancient skeleton attained over 30 pM of DNA, and after dilution, 30 pM of DNA was used in MPS according to the manufacturer’s recommendations [71].

The main sequencing parameters of the bone samples from the adult and subadult ancient skeleton showed that the libraries yielded from 55.2% (adult skeleton) to 91.6% (subadult skeleton) on-target reads, the uniformity of sequencing was 100% for both skeletons, the average mapped reads values were 513,387 for the adult skeleton and 515,047 for the subadult skeleton, and the mean depth values were 14,574 for the adult skeleton and 25,636 for the subadult skeleton. When using the settings from Converge software (TFS), all the reads exceeded the threshold of 20 × . Coverage values for each HIrisPlex SNP marker and for each nucleotide variant detected by MPS in each of the markers, together with the genotypes for both skeletons analyzed, are shown in SM 3. From the adult skeleton, a HIrisPlex genotype with one missing SNP marker was obtained (NN was attributed to SNP marker rs201326893), and the subadult skeleton generated a complete HIrisPlex genotype (see SM 3). The HIrisPlex-S panel validation study [6] sets the recommended coverage value thresholds for each SNP marker for samples above and below 100 pg DNA input. In the adult and subadult ancient skeletons, the MPS coverage values significantly exceeded the recommended thresholds for > 100 pg DNA input, as expected for samples in which the optimal template DNA amount of 1 ng was used for amplification in HIrisPlex PCR-MPS analysis. For the ancient adult and subadult skeleton samples, no heterozygous or homozygous genotypes were flagged by the Converge software (TFS), showing no unambiguous results, except for rs201326893 missing data for the adult skeleton.

Eye and hair color were predicted from genotypes obtained from the adult and subadult skeleton using the HIrisPlex web tool and the user manual for HIrisPlex (https://hirisplex.erasmusmc.nl/). Table 1 presents the *p*-values for various eye colors, hair colors, and hair shades, and AUC loss values and the most probable phenotype predicted for both skeletons studied are shown.

**Table 1:** Results of eye and hair color prediction for the ancient adult and subadult skeleton together with *p*-values for eye colors, hair colors, and hair shades; AUC loss values; and the most probable predicted phenotype

		Probability values (*p*-value)		AUC loss		Most likely predicted color category	
		Adult	Subadult	Adult	Subadult	Adult	Subadult
Eye colour	Blue eye	0.033	0.876	0	0	Brown eyes	Blue eyes
	Intermediate eye	0.108	0.090	0	0		
	Brown eye	0.859	0.035	0	0		
Hair color	Blond hair	0.031	0.350	0.001	0	Dark brown/black hair	Brown/dark brown hair
	Brown hair	0.590	0.571	0	0		
	Red hair	0.000	0.004	0.003	0		
	Black hair	0.379	0.075	0	0		
Shade	Light hair	0.146	0.817	0	0		
	Dark hair	0.854	0.183	0	0		

The AUC loss in the subadult sample extract was 0 because there were no allelic drop-outs, and a complete HIrisPlex genotype was generated. Because of missing data for amplification of SNP marker rs201326893 in the adult skeleton, AUC loss was calculated for blond hair and red hair, and the highest was for red hair color prediction (0.003).

In the adult skeleton, the highest *p*-value was 0.859 for brown eyes, and in the subadult skeleton, it was 0.876 for blue eyes. For hair color prediction, the *p*-value for brown hair was 0.59 and for light hair value, it was 0.146, resulting in a dark brown/black hair color prediction for the adult skeleton. For the subadult skeleton, the *p*-value for brown hair was 0.571, and for light hair value, it was 0.817, resulting in a brown/dark brown hair color prediction. A phenotype of brown eyes and dark brown or black hair was predicted for the adult ancient skeleton, whereas the most probable hair color for the subadult ancient skeleton was brown or dark brown, and the eye color was blue (see Table 1).

## Discussion

Partial or incomplete HIrisPlex profile generation is expected for ancient samples because low amounts of poor-quality DNA are usually obtained from them. Surprisingly, full or almost full profiles were obtained not only for the adult skeleton but also for the subadult skeleton from the Early Middle Ages, and hair and eye colors were successfully predicted even though some AUC losses were calculated for prediction of the hair color of the adult skeleton. We have to be aware that Table 1 shows the most probable phenotypes predicted using the HIrisPlex tool, and developmental validation study of the HIrisPlex System showed overall prediction accuracies for eye color at 0.94 for blue, 0.74 for intermediate, and 0.95 for brown, and for hair color at 0.81 for blond, 0.75 for brown, 0.92 for red, and 0.85 for black hair with a hair color shade at 0.90 [78]. When the HIrisPlex prediction tool was used on a set of 119 individuals from the Polish population, eye color was predicted with 84% accuracy, and hair color with 73% accuracy on average, meaning that in three out of four individuals, prediction was accurate [78]. None of the *p* values for eye and hair color prediction was above the accuracy threshold values in the adult and subadult skeleton analyzed, showing that regardless of whether we are testing recent individuals or ancient skeletons, DNA phenotyping is a probability-based prediction, and the results are based on knowledge from a combination of genotypes from thousands of individuals included in a model used in the HIrisPlex prediction tool. An anomaly with the subadult skeleton having a high probability for light hair, but a conclusion of brown or dark brown hair shown in Table 1, is presumably also related to lower probability values that do not reach the accuracy thresholds. However, it would be interesting to compare eye and hair color frequencies in the contemporary Slovenian population with the predicted phenotypes of the ancient adult and subadult skeleton from the fifth- to sixth-century Bled–Pristava archaeological site. In 2012, eye and hair color frequencies of the contemporary Slovenians were published, and for eye color, 44.7% blue, 25.7% intermediate, and 29.6% brown color were observed, whereas hair color was defined as blond in 5.7% of Slovenians, dark blond or light brown in 41%, and dark brown or black in 52.4% [37]. Unfortunately, no information on the combination of both phenotypes is provided for the contemporary Slovenian population, and information on how common phenotypes are with a combination of brown eyes and dark brown or black hair (predicted for the adult skeleton) and blue eyes and brown or dark brown hair (predicted for the subadult skeleton) is not available. The combination of blue eyes and brown or dark brown hair seems to be a less common phenotype in the Slovenian population. However, when several ancient and Second World War skeletons from Slovenia were used for eye and hair color prediction, in two skeletons, dark hair was predicted using the HIrisPlex tool despite the predicted blue color phenotype for the eyes. The ancient skeleton from the sixteenth century had a high probability for brown or dark brown hair and the Second World War skeleton even for dark brown or black hair [83]. Genetic similarities of skeletons excavated from the multiple burial grave from the Bled–Pristava archaeological site to contemporary Slovenians were confirmed in our previous study through Y chromosomal STR markers, and E1b1b haplogroup was predicted [88]. The same haplogroup was detected with a frequency of 7.3% in the current Slovenian population [81]. As expected (because of the very high variability of autosomal STR markers), the STR genotypes of the adult and subadult skeleton showed low frequencies in the contemporary Slovenian population. As it was shown, modern forensic genetics methods can interact and enhance modern archeology, not only through confirmation of kinship between individuals from the same archaeological site [88] and through searching for genetic similarities to the contemporary human populations, but also through prediction of phenotypic traits to obtain information about the external appearance of adult and subadult individuals from past human populations.

A search for pigmentation trait prediction in ancient subadult skeletons did not yield any studies on genetic prediction of eye and hair color for subadults. The reasons for this lie in the small number of subadult skeletons represented in archaeological osteological collections and in the poor preservation of DNA in subadult skeletons compared to adult skeletons. Namely, in archaeology, subadults are very often underrepresented in collections of excavated skeletons due to their bones’ suboptimal survival rate [10, 27, 36, 45, 66, 67]. Compared to adults, subadults have greater susceptibility to environmental factors affecting DNA preservation [3, 31, 52, 66], and, because of their smaller, more porous, and less dense bones, subadult skeletons are more prone to decay [14, 27, 50, 75, 76, 80]. In addition, subadult bones have lower inorganic and higher organic content, resulting in less preserved DNA in comparison to adult skeletons [73].

The results of a study performed by Šuligoj et al. [70], which analyzed adult and subadult ancient skeletons dating to the seventeenth to nineteenth century, showed that among the various skeletal element types analyzed (femurs, tali, calcanei, and petrous bones were studied), only the petrous bones yielded amplifiable amounts of DNA in subadult skeletons. In contrast, in adult skeletons, amplifiable DNA yields were obtained not only from petrous bones but also from tali and calcanei, whereas femurs showed low DNA yields and low STR typing success. Petrous bones proved to be the only suitable source of DNA for aDNA analysis of subadult skeletons (when compared to tali, calcanei, and femurs), and, interestingly, DNA yields were even higher than in the adult petrous bones, which the authors attributed to the higher degradation of DNA measured in adult petrous bones [70]. In the case of the adult and subadult early medieval skeletons analyzed in this study, similar DNA yields were observed from both skeletons, but nonetheless, amplification of HIrisPlex SNPs was more successful in the subadult than adult skeleton, obtaining a full HIrisPlex genotype with higher coverage values in all SNPs typed in the subadult skeleton and partial genotype—with one HIrisPlex SNP missing—and lower coverage values in the adult skeleton in spite of the fact that 1 ng of DNA was used for HIrisPlex PCR-MPS analysis for both petrous bones. In addition, in the adult skeleton, the percentage of on-target reads was lower than in the subadult skeleton (55% versus 91%). The difference in sequencing parameters and typing performance is probably the result of differences in DNA degradation because the degradation index in the adult skeleton was higher than in the subadult skeleton. A similar difference in DNA degradation rate was observed in the study performed by Šuligoj et al. [70], in which the DNA quantity and quality of 20 ancient adult and 20 ancient subadult petrous bone extracts were compared, and greater degradation of DNA was observed in adult petrous bones than in subadult ones. The reasons for the less-degraded DNA observed in the ancient subadult petrous bone in comparison to the ancient adult petrous bone, even though they were both exposed to similar environmental factors for the same amount of time, are unknown. The petrous bone is characterized by unique properties and completes its development in the embryonic stage. In the 16th week of gestation, when the cartilage labyrinth reaches its final size, ossification begins and is completed by birth [46]. The petrous bone has an atypical structure and unique tissue organization, with very dense, thick, compact bone, high cellularity, and low trabecular bone content [13, 15, 35, 43]. Because of a lack of remodeling, the petrous bone is characterized by its immature nature, and its limited vascularization inhibits contamination by exogenous microorganisms [24]. The petrous bone’s high density improves its resistance and decreases bacterial DNA decomposition, damage, and degradation [59]. The protective shell that surrounds the inner ear additionally promotes the preservation of endogenous DNA in the petrous bone [24]. As shown previously by various aDNA studies, it was also proved in the study of eye and hair color prediction for the early medieval adult and subadult skeleton presented here that petrous bones generate high-quality HIrisPlex genotypes in adult and subadult skeletons.

Because not only HIrisPlex SNPs were typed, but STR typing was also performed on the ancient adult and subadult skeletons, it was possible to determine the sex of the skeletons analyzed. Morphological methods to assess subadults’ sex are not sufficiently accurate and are not advisable at this time [9, 69]. Some research has acknowledged the strong potential of DNA testing for increasing the precision of morphological sex assessment [2, 26, 33, 47, 54], which was also clear in the study of the adult and subadult skeletons from the Early Middle Ages, for which anthropological analysis was only able to assess the sex of the adult skeleton, whereas determination of sex could not be made for the subadult skeleton. Molecular genetic methods corroborated the adult skeleton’s anthropologically defined male sex, and the sex of the subadult skeleton was also successfully established through successful qPCR PowerQuant Y target amplification and Y chromosome amelogenin amplification. Male sex was determined for the adult and subadult skeletons.

Because this study was carried out on ancient skeletons, measures to prevent contamination were necessary [55, 84]. Both skeletons were typed for autosomal STRs in duplicate (from different extracts obtained from the same bone) to verify the genetic typing results. We carried out STR typing of the personnel in the elimination database, and STR analysis was used to monitor the purity of ENCs [57]. ENCs were examined only for STR markers because of the expense of the MPS reagents. When the adult and subadult petrous bones were analyzed, there was no contamination of ENCs, which yielded no qPCR or STR typing results. Moreover, no match occurred between the STR profiles of the bones analyzed and the profiles in the elimination database. In addition, autosomal STR matches of duplicated STR analysis of both extracts obtained from the petrous bones also confirmed the absence of contamination events. Degradation rate is utilized as a criterion for the authenticity of ancient DNA [55], and in both skeletons analyzed, high degradation was proved (with much lower copy numbers of Deg than Auto target, as determined through PowerQuant quantification).

## Conclusions

The results of this study have confirmed successful HIrisPlex-based PCR-MPS eye and hair color prediction for early medieval skeletons. As already shown in various aDNA studies, it was proved again that petrous bones are an excellent source of DNA for various genetic analyses. Exceptional preservation of DNA in petrous bones resulted in generation of high-quality HIrisPlex genotypes not only in an adult skeleton but also in a subadult skeleton dated to the fifth to sixth century. Moreover, sequencing parameters and genotyping performance were even higher in the subadult skeleton than in the adult skeleton. Studies on pigmentation trait prediction performed on subadult ancient skeletons are very rare, and so the results of this study may be helpful to forensic experts when it is necessary to predict eye and hair color in missing person identification cases when aged subadult skeletal remains are found and must be identified, but there are no clues regarding who the missing child might be. By predicting the hair and eye color, the circle of missing children can be narrowed, and investigation can be directed to successful identification. In the case of forensic subadult skeletal remains, better DNA preservation can be expected than in ancient skeletons due to shorter exposure of the remains to harmful environmental influences, and petrous bone sampling is recommended for phenotyping.

## Supplementary information

Below is the link to the electronic supplementary material.

Supplementary file1 (DOCX 18 KB)

Supplementary file2 (XLSX 14 KB)

Supplementary file3 (XLSX 16 KB)

## Data Availability

The authors declare that all the data are available.
