# Supplementary material for: Eye and hair color prediction of an early medieval adult and subadult skeleton using massive parallel sequencing technology
Source: Int J Legal Med. 2023 Jun 7;137(5):1629–38. doi: 10.1007/s00414-023-03032-y (PMC10421759; doi:10.1007/s00414-023-03032-y)
Supplement: Supplementary file 1 — Supplementary file1 (DOCX 18 KB) [file 414_2023_3032_MOESM1_ESM.docx]

Table SM 1: Characteristics (skeleton, skeletal type and extraction number, extraction negative control - ENC), PowerQuant results (DNA quantity - Auto target, Deg target and Y target - all expressed in ng DNA in µl of extract , IPC shift and degradation index - DI), and DNA quantity expressed in ng DNA per g of bone powder for adult and subadult skeleton from the Early Middle Ages.

| Skeleton | Skeletal type | DNA quantity-AUTO target (ng/µl) | DEG target  (ng/µl) | Y target  (ng/µl) | IPC shift | Deg.  Index (DI) | DNA quantity (ng/g powder) |
| --- | --- | --- | --- | --- | --- | --- | --- |
| Adult | petrous bone  (extract 1) | 0,2105 | 0,0007 | 0,1005 | -0,24 | 305,02 | 21,05 |
| Adult | petrous bone  (extract 2) | 0,1379 | 0,0006 | 0,0506 | -0,40 | 247,40 | 13,79 |
| Subadult | petrous bone  (extract 1) | 0,2145 | 0,0296 | 0,0849 | -0,04 | 7,26 | 21,45 |
| Subadult | petrous bone  (extract 2) | 0,1170 | 0,0012 | 0,0533 | 0,02 | 99,43 | 11,70 |
|  | ENC 1 | / | / | / | -0.34 | / | / |
|  | ENC 2 | 0,0002 | / | / | -0.24 | / | 0,02 |
